# The use of whole body computed tomography does not lead to increased 24-h mortality in severely injured patients in circulatory shock

**DOI:** 10.1038/s41598-024-52657-5

**Published:** 2024-01-25

**Authors:** Ivana Hanzalova, Mylène Bourgeat, Nicolas Demartines, François-Xavier Ageron, Tobias Zingg

**Affiliations:** 1 Department of Surgery, Lausanne University Hospital (CHUV) and Lausanne University, Rue du Bugnon 46, 1011 Lausanne, Switzerland; 2 Department of Emergency Medicine, Lausanne University Hospital (CHUV) and Lausanne University, Rue du Bugnon 46, 1011 Lausanne, Switzerland

**Keywords:** Diseases, Health care, Medical research

## Abstract

The Advanced Trauma Life Support (ATLS) approach is generally accepted as the standard of care for the initial management of severely injured patients. While whole body computed tomography (WBCT) is still considered a contraindication in haemodynamically unstable trauma patients, there is a growing amount of data indicating the absence of harm from cross sectional imaging in this patient group. Our study aimed to compare the early mortality of unstable trauma patients undergoing a WBCT during the initial workup with those who did not. Single-center retrospective observational study based on the local trauma registry including 3525 patients with an ISS > 15 from January 2008 to June 2020. We compared the 24-h mortality of injured patients in circulatory shock undergoing WBCT with a control group undergoing standard workup only. Inclusion criteria were the simultaneous presence of a systolic blood pressure < 100 mmHg, lactate > 2.2 mmol/l and base excess < − 2 mmol/l as surrogate markers for circulatory shock. To control for confounding, a propensity score matched analysis with conditional logistic regression for adjustment of residual confounders and a sensitivity analysis using inverse probability weighting (IPW) with and without adjustment were performed. Of the 3525 patients, 161 (4.6%) fulfilled all inclusion criteria. Of these, 132 (82%) underwent WBCT and 29 (18%) standard work-up only. In crude and matched analyses, no difference in early (24 h) mortality was observed (WBCT, 23 (17.4%) and no-WBCT, 8 (27.6%); p = 0.21). After matching and adjustment for main confounders, the odds ratio for the event of death at 24 h in the WBCT group was 0.36 (95% CI 0.07–1.73); p = 0.20. In the present study, WBCT did not increase the risk of death at 24 h among injured patients in shock. This adds to the growing data indicating that WBCT may be offered to trauma patients in circulatory shock without jeopardizing early survival.

## Introduction

Trauma is one of the major contributors to the global burden of death and permanent disability, with road and self-harm injuries being the leading causes among the population from 10 to 49 years of age^1^. Currently, about one third of post-traumatic deaths occur before hospital admission or within the first 24 h thereafter^2^. Thus, efforts aiming to decrease trauma mortality must focus on primary prevention, early identification and treatment of life-threatening injuries, such as tension pneumothorax and/or significant bleeding^3^. The Advanced Trauma Life Support^4^ (ATLS) course serves as a globally established, systematic clinical guideline for the initial evaluation and treatment of the severely injured. For trauma patients in shock, extended focused assessment with sonography for trauma (eFAST) and conventional radiographs (chest, pelvis) are ATLS-recommended imaging adjuncts to help rapidly localize the source of circulatory instability during the primary exam. ATLS recommends cross-sectional imaging with computed tomography only in haemodynamically stable trauma patients^4^.

The use of whole-body computed tomography (WBCT) has increased in the initial assessment of injured patients during the last decade. WBCT allows for a fast and complete overview of injuries and identification of the source of bleeding. Several observational studies have shown a reduction in the risk of mortality for trauma patients who underwent WBCT compared to standard work-up with radiographs and ultrasonography, with or without selective CT^5–7^. However, one randomised controlled trial, the REACT-2 study, showed no differences in mortality between WBCT and standard work-up imaging with additional selective CT. A recent meta-analysis showed a non-significantly lower in-hospital mortality^8^. However, they found a reduction in the time spent in the emergency department in favour of WBCT. Recent European guidelines recommend the use of early WBCT for the detection of the source of bleeding^9^. However, due to a gap in the evidence concerning the benefit of WBCT in all severely injured patients and concerns with potentially harmful radiation exposure, some authors recommend a tailored approach with selective use of WBCT in injured patients^10^.

Although more sensitive and specific than ultrasound and radiographs for identifying traumatic injuries^11^, WBCT is currently not recommended in case of haemodynamic instability, to avoid circulatory arrest from ongoing bleeding during its more time-consuming image acquisition phase. With advanced techniques and faster WBCT protocols, this “dogma” has been repeatedly challenged. Several studies have suggested the safety of WBCT and a survival benefit in unstable trauma patients^12–17^. A second analysis of the REACT-2 trial showed that WBCT led to a strong reduction in mortality in injured patients requiring emergency bleeding control interventions, but failed to demonstrate a statistical significance due to an underpowered study^10,18^. However, in all these studies, the definition of haemodynamic instability was based on vital signs (systolic blood pressure, heart rate, respiratory rate, Glasgow Coma Scale score) without physiological surrogates of shock leading to heterogeneity in the treatment effect.

There is no universal definition of haemodynamic instability. Systolic blood pressure and heart rate with various cut-offs at different time points, are the most commonly used parameters leading to frequent misclassification^19^. Vital signs alone cannot be used as surrogate markers for tissue perfusion. More contemporary definitions of haemodynamic instability therefore include metabolic parameters such as base deficit and lactate^20–22^ therefore decreasing the risk of erroneously including solely hypotensive or tachycardic patients without evidence of shock as haemodynamically unstable in retrospective cohort studies.

The aim of our study was to analyse whether performing a WBCT is associated with early mortality among trauma patients with physiological evidence of circulatory shock, defined as the simultaneous presence of SBP < 100 mmHg, lactate > 2.2 mmol/l and base excess < − 2 mmol/l.

## Methods

### Study design and setting

We performed a retrospective observational study with propensity score-matching analysis based on the data from a single Swiss tertiary trauma center. Our tested hypothesis was that there would be no difference in mortality if WBCT was used during the initial work-up in the resuscitation room in injured patients in circulatory shock.

Lausanne University Hospital is a tertiary hospital and one of 12 designated trauma centers in Switzerland, with full diagnostic and interventional capabilities 24/7. Trauma care is guided by current clinical recommendations, internally adapted to the local infrastructure and clinical expertise. Severely injured patients are being cared for in prepared shock rooms of the emergency department by a dedicated team composed by two ED physicians, two ED nurses, one anaesthesiologist, one anaesthesia nurse, one general surgeon, one orthopaedic trauma surgeon and a neurosurgeon, with consultants from all other specialties available 24/7.

The Lausanne University Hospital trauma protocol follows current ATLS principles adapted to the local infrastructure with a CT scanner, located next to the emergency department's resuscitation room. The institutional polytrauma CT protocol was performed with a 64-detector row CT (Light Speed VCT 64 Pro; GE Healthcare, Milwaukee, WI, USA). 1.25 mm reconstructed axial slices with increments of 1 mm were obtained during the arterial phase (25s) centred on the thorax, and 2.5 mm reconstructed axial slices with increments of 2 mm were obtained during the venous phase (80s) centred on the abdomen and pelvis, after intravenous injection of iodinated contrast medium Accupaque^®^ at a flow rate of 4 ml/s [120 kV, 300 mA, table speed 55 mm per rotation (0.8s), pitch 1.375]. Automatic tube current modulation in all three axes (SmartmA) was used as well as iterative reconstruction algorithm ASIR. The decision to proceed with WBCT depends on the trauma leader—either a senior general surgeon or a senior emergency physician.

### Population

**Figure 1:**
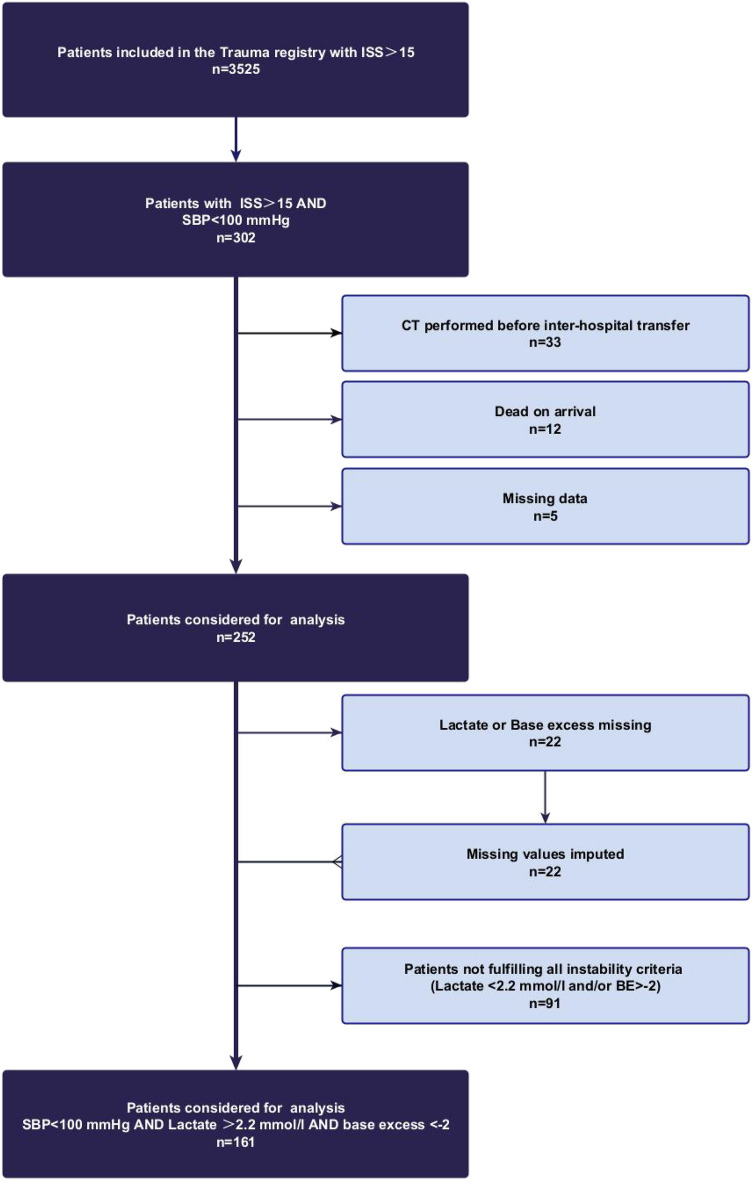
Study flowchart.

We selected injured patients in circulatory shock included in the Trauma Registry of Acute Care (TRAC) between 1.1.2008 and 30.06.2020. During the study period, a total of 3525 patients with an ISS > 15 were admitted. Circulatory shock was defined as the simultaneous presence of an initial systolic blood pressure (SBP) < 100 mmHg, blood lactate > 2.2 mmol/l and base excess < − 2 mmol/l, based on contemporary recommendations integrating metabolic surrogate markers of tissue hypoperfusion such as lactate and Base excess rather than solely arterial hypotension^20–22^. The cut-off values for lactate (> 2.2 mmol/l) and base excess (< − 2 mmol/l) were chosen based on the normal ranges of our institution’s laboratory. The chosen cut-off level for lactate at 2.2 mmol/l is in accordance with, or at least extremely similar to, the recommendations of the European Society of Intensive Care Medicine (> 2 mmol/l)^22^. Base excess was used in combination with lactate as a quantitative marker for metabolic acidosis and to exclude cases with elevated lactate levels from other causes than tissue hypoperfusion (muscular injury, drugs, pharmacological agents). When lactate or base excess were missing, these values were imputed with multiple chained equation (MICE) to avoid selection bias. Exclusion criteria were cardiac arrest upon arrival, CT performed in another hospital, burn patients, age < 16 years. Figure 1 illustrates the study inclusion flowchart.

### Outcomes and intervention

We compared early mortality at 24 h as a primary outcome between two groups of patients in circulatory shock being worked up either with the classical ATLS approach alone or with WBCT. Secondary outcomes were as follows: mortality at 30 days, length of intensive care unit (ICU) stay, overall length of stay, and time to lactate clearance.

The WBCT exam during the initial work-up in the resuscitation room was the intervention in the current study. Patients not undergoing WBCT but only standard radiologic workup according to the ATLS guidelines (chest and pelvis X-ray, eFAST) were matched to the group undergoing WBCT.

### Statistical analysis

Categorical variables are described as count and percentage. Continuous variables are described as mean and standard deviation (sd) or median and interquartile range (IQR) depending on the normal or skewed distribution. Categorical variables were compared by either Chi-2 test or Fisher exact test. Continuous variables were compared by either Student T test or Wilcoxon rank sum test depending on the parametric or nonparametric distribution.

As patients were not randomly assigned to WBCT, we performed propensity score (PS) matching analysis to limit unknown confounders. The PS is the probability that each patient will be placed in one of the groups to receive, or not, the treatment of interest given their measured covariates. We developed a directed acyclic graph (DAG) to select the variables used in the propensity score (Supplementary file 1). We included age, BATT (Bleeding Audit and Triage Trauma) score (estimating the baseline risk of death from bleeding), AIS head score, ISS, injury mechanism (penetrating injury, high energy trauma), GCS and SBP on arrival, ASA score, positive e-FAST, ongoing anticoagulation treatment, massive transfusion and any pelvic ring injury on X-ray. High energy trauma was defined as either a road traffic crash (with intrusion, ejection, death in the same passenger compartment, and motor vehicle versus pedestrian or bicyclist), a fall from a height > 3 m, or a blow or blast injury. These indicators were chosen to ensure comparison of primary and secondary outcomes between patients with similar pre-injury characteristics and injury severities and to identify potential confounding variables. All available clinical, laboratory, and imaging results were obtained and recorded during the initial phase of care in the ED.

The BATT score has been previously validated as a prediction tool for mortality due to haemorrhage in trauma patients^23^. The ASA score was determined based on patient data from the electronic healthcare record^24^. Positive e-FAST was defined as free intraperitoneal, pleural or pericardial fluid or absence of lung sliding^25^. Chest X-ray was considered positive in case of pneumothorax, haemothorax or serial rib fractures (3 or more consecutive ribs)^26^. Massive transfusion was defined as receiving ≥ 10 units of whole blood (WB) or red blood cells (RBCs) within the first 24 h^27^. Propensity score matching was performed with a matching ratio of one patient without WBCT to 5 with WBCT, with a low matching calliper of 0.05 in order to pair similar physiological and injury characteristics on arrival between the two groups. We compared mortality at 24 h and at 30 days in crude and propensity score matching analysis. We performed a multivariate analysis with a conditional logistic regression model in the matched cohort. We adjusted for known confounders (ISS, GCS, BATT, ASA, AIS head, positive X-ray, positive FAST). In addition, we performed an inverse probability weighting (IPW) analysis (crude and adjusted). IPW performed analysis by weighting the outcome by the inverse of the probability to be assigned in the WBCT group (PS). We plotted all the risk differences estimated by the different analyses in the same graph. All analyses were used as sensitivity analysis to one another.

Statistical analyses and graphics were performed using STATA software version 16 (Stata Corp., College Station, TX, USA). Psmatch2 package was used for propensity score matching analysis. The proportion of missing values is described in the results section. Missing values represented less than 2% of the data. We imputed (using multiple chained equation) blood lactate and Base excess, GCS, Heart rate, Hemoglobin level and Respiratory rate when missing.

The sample size was fixed due to the retrospective registry-based design. The power of the study was estimated by post-hoc calculation.

The local ethics committee (CER-VD) approved the study protocol (No. 2020-01238) and waived the need for informed consent. All methods were performed in accordance with the local ethics and institutional guidelines and regulations and the manuscript was prepared to conform to the STROBE guidelines^28^.

### Ethics approval and consent to participate

The study was registered as No. 2020-01238 and approved by local ethics comitee (Commission Cantonale d'Éthique de la Recherche sur l'Être Humain Vaud (CER-VD).

## Results

### Descriptive analysis

During the study period, 3525 severely injured patients were admitted, of which 302 (8.6%) had a SBP < 100 mmHg and 161 (4.6%) met all inclusion criteria. Among the latter, 132 (82%) underwent WBCT. The baseline characteristics of the unmatched and propensity score matched cohorts are summarized in Table 1. Due to an excessively high or low PS, rendering matching impossible, 14 patients were subsequently not included in the matched cohort.

**Table 1:** Patient characteristics.

	Missing (%)	Unmatched cohort N = 161			PS-matched cohort N = 147		
		CT	no CT	p value	CT	No CT	p value
Pre-injury							
Age, mean (SD)	0	49 (21)	42 (19)	0.12	48 (20)	47 (29)	0.79
Sex female, N (%)	0	35 (27)	8 (28)	0.91	32 (27)	7 (26)	0.89
Relevant drug treatement^a^, N (%)	0	8 (7)	2 (7)	0.98	8 (7)	1 (4)	0.38
ASA pre-injury, median [IQR]	2	2 [1, 2]	2 [1–3]	0.08	2 [1, 2]	2 [1–3]	0.24
Prehospital							
Intubated before ED arrival, N (%)	15	44 (33)	7 (24)	0.25	37 (31)	9 (33)	0.67
BATT score, mean (SD)	6	11 (4)	13 (5)	0.03	11 (4)	11 (4)	0.31
ED							
SBP, mean (SD)	0	82 (20)	69 (29)	< 0.01	81 (21)	79 (20)	0.58
Lactate, mean (SD)	–	6 (5)	9 (6)	< 0.01	6 (4)	7 (5)	0.11
BE, mean (SD)	–	− 12 (8)	− 15 (8)	0.06	− 12 (9)	− 12 (7)	0.62
GCS, mean (SD)	2	8 (5)	8 (6)	0.80	8 (5)	7 (7)	0.09
MGAP score, mean (SD)	2	15 (6)	15 (8)	0.84	15 (6)	15 (9)	0.59
Shock Index, mean (SD)	9	1.3 (0.5)	1.4 (0.5)	0.30	1.3 (0.5)	1.3 (0.5)	0.96
Time from ED arrival to CT (min), median [IQR]	0	32.5 [25–47]	–	–	33.5 [24–49]	–	–
Trauma characteristics							
ISS, mean (SD)	0	27 (16)	27 (16)	0.96	25 (15)	24 (17)	0.87
Penetrating, N (%)	0	11 (8)	7 (24)	0.01	11 (9)	8 (28)	< 0.001
AIS Head ≥ 3, N (%)	0	49 (37)	7 (24)	0.18	36 (30)	13 (48)	0.01
Standard ED imaging							
FAST pos., N (%)	14	23 (17)	13 (45)	0.001	22 (19)	7 (28)	0.10
Chest x- ray pos., N (%)	1	47 (36)	12 (41)	0.56	39 (33)	8 (29)	0.52
Pelvic x-ray pos., N (%)	1	25 (19)	12 (41)	0.01	25 (21)	8 (29)	0.15
Treatment							
Surgery, N (%)	0	54 (41)	20 (69)	0.01	49 (42)	18 (68)	< 0.001
Angio-embol., N (%)	0	13 (10)	2 (7)	0.62	13 (11)	1 (2)	0.01
Conservative, N (%)	0	65 (49)	7 (24)	0.01	56 (47)	8 (29)	0.004
ICU admission, N (%)	0	92 (70)	16 (55)	0.13	79 (67)	16 (58)	0.17

Coumarine derivatives, platelet inhibitors and NOACs.

### Unmatched cohort

Before matching, the WBCT group had significantly lower risk of death from bleeding BATT scores (11 vs 13, p = 0.03), lactate levels (6 vs 9 mmol/l, p = 0.001), rates of penetrating trauma (8% vs 24%, p = 0.001), positive FAST exams (17% vs 45%, p = 0.001), positive pelvic radiographs (19% vs 41%, p = 0.01) and rates of surgical management (41% vs 69%, p = 0.01). Systolic blood pressures were higher (82 mmHg vs 69 mmHg, p < 0.01), and non-operative management was more often performed (49% vs 24%, p = 0.01) in the WBCT group.

### Matched cohort

After propensity score matching, there were less patients with severe head injury (AIS ≥ 3) in the WBCT group (30% vs 48%, p = 0.01). The WBCT group still had lower rates of penetrating trauma (9% vs 28%, p < 0.001), and surgical management (42% vs 68%, p < 0.001), while non-operative management remained more frequent (47% vs 29%, p = 0.004). The rate of management with angio-embolisation was also higher in the WBCT group (11% vs 2%, p = 0.01) after matching.

**Figure 2:**
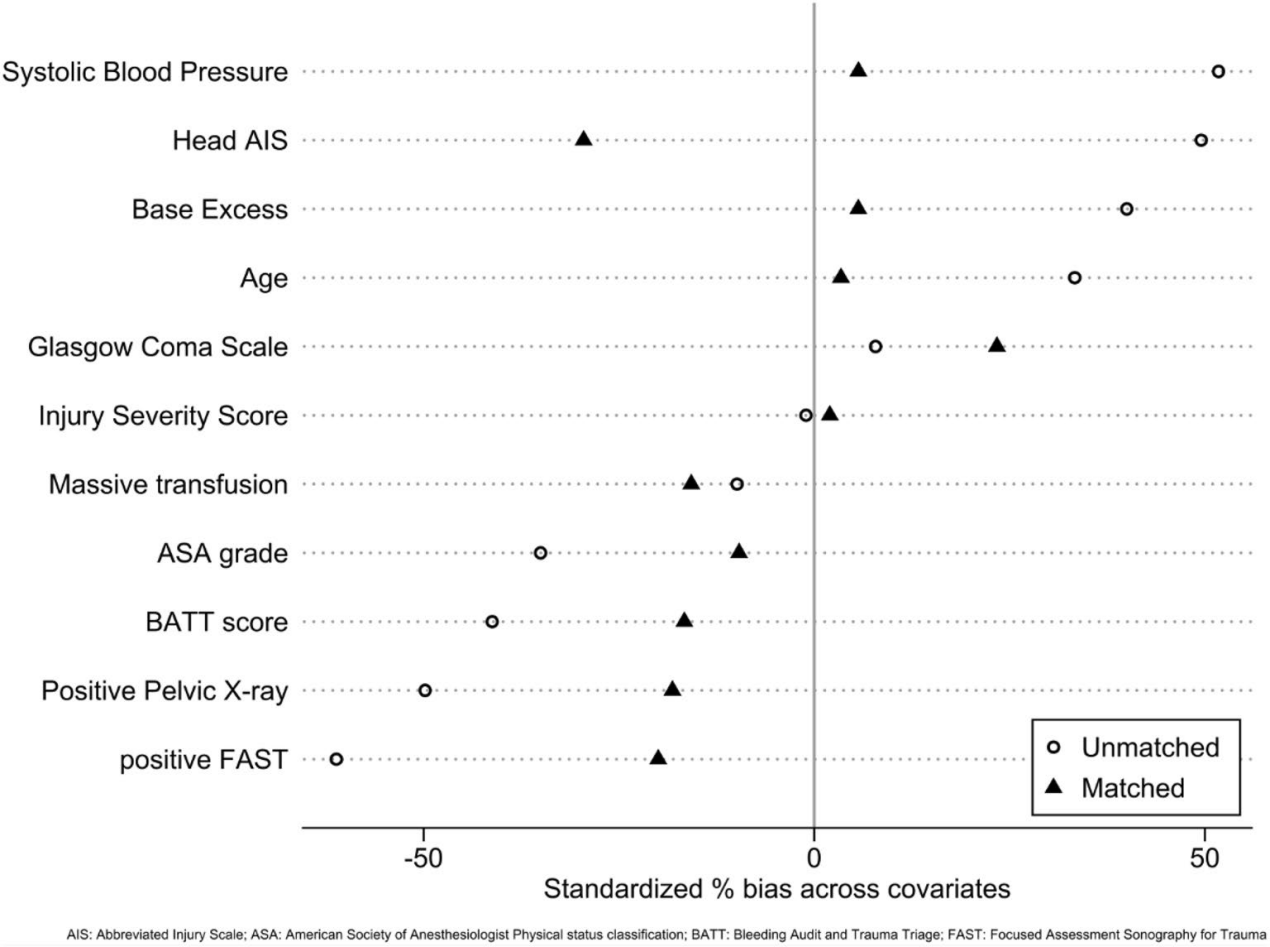
Effect of Propensity Score matching on bias reduction on co-variates.

Figure 2 illustrates the effect of propensity score matching on bias reduction among the covariates. The standardised bias was reduced from 38.7% to 13.5% with a reduction in R_2_ from 0.025 to 0.003.

### Outcome analysis

Table 2 summarises the results for the primary and secondary outcomes.

**Table 2:** Primary and secondary outcomes.

	Unmatched cohort n = 161			PS-matched cohort n = 147		
	CT	No CT	p value	CT	No CT	p value
Primary outcome						
24-h mortality, N (%)	23 (17)	8 (28)	0.21	20 (17)	7 (26)	0.59
Secondary outcomes						
In hospital (30 days) mortality, N (%)	38 (29)	9 (31)	0.81	30 (25)	5 (20)	0.68
Median overall length of stay, days [IQR]	9 [1–22]	9 [0–29]	0.83	10 [1–26]	9 [0–29]	0.63
Median ICU stay, days [IQR]	1 [0–11]	1 [0–6]	0.94	1 [0–6]	1 [0–11]	0.95
Massive transfusion, N (%)	18 (14)	5 (17)	0.62	17 (14)	5 (20)	0.24
Median time to clear lactate, hours [IQR]	17 [5–167]	16 [6–167]	0.95	14 [5–167]	16 [6–167]	0.93

Before PS matching, the primary outcome of 24-h mortality occurred in 23 (17%) patients who had undergone WBCT and 8 (28%) among patients with standard radiologic workup (p = 0.21). The crude absolute risk reduction of early death was 10% with 95% CI (− 26; + 6) in favor of WBCT compared to the standard work-up (p = 0.21).

The propensity-score matched analysis showed a 24-h mortality of 17% and 26% in the WBCT and standard groups respectively (p = 0.60), and a non-significant risk reduction of early death of 9% (95% CI – 33 to + 15), in favor of WBCT (p = 0.60). This corresponds to an adjusted odds ratio for death at 24 h in the WBCT group of 0.36 (95% CI 0.07–1.73; p = 0.20). There were no differences in secondary outcomes between the two groups.

**Figure 3:**
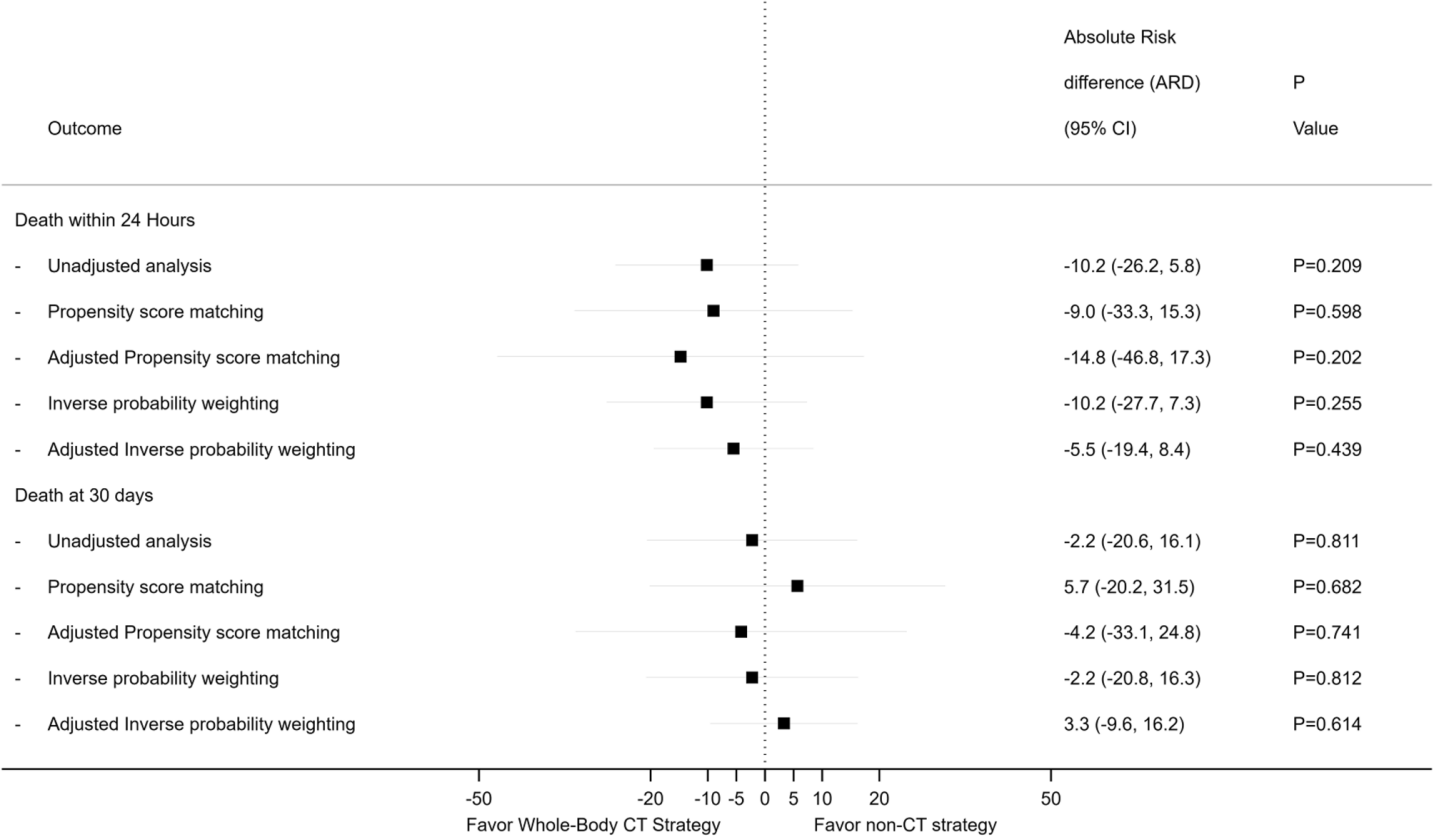
Forrest plot: average treatment effect of WBCT on mortality, displayed as absolute risk difference.

Figure 3 illustrates the absolute risk difference for early and late death between WBCT and standard workup, by method of analysis: crude unadjusted, adjusted and non-adjusted propensity score matching, and adjusted and non-adjusted inverse probability weighing. Early and late mortalities were not statistically different between the WBCT and the standard work-up groups.

The post-hoc calculation based on propensity-score matching analysis showed a power of 22%.

Crude analysis of the data excluding patients with a penetrating injury mechanism resulted in a significant difference in favour of WBCT, with an early death rate of 17.3% in the WBCT group versus 36.4% in the no-CT group (p = 0.04). In the matched analysis however, this difference (17.4% versus 33.3%) was no longer significant (p = 0.42).

## Discussion

The present study did not find any association between WBCT and mortality within 24 h and at 30 days in injured patients in circulatory shock. Most of the patients underwent a WBCT during their initial management in the emergency department. Their 24-h mortality was not increased compared to a propensity score matched control group undergoing the currently recommended standard work-up without WBCT. Even though not statistically significant, the group undergoing a WBCT had an absolute risk reduction for early mortality of 9–10%.

WBCT has the advantage of being highly sensitive and specific for identifying a source of significant bleeding, which can then guide surgery, non-operative management or interventional radiology (stenting for traumatic aortic dissection, embolisation of acute splenic or liver haemorrhage)^29^. There is an evolving concept of damage control interventional radiology for unstable trauma patients^30^. WBCT can identify non-haemorrhagic causes of haemodynamic instability, such as intra-cranial or spinal cord injury, thereby directing therapy and preventing non-therapeutic surgical exploration of the chest or the abdomen.

Our results are in line with findings from several previous studies. Like most of the literature on WBCT,we used early mortality as the primary outcome. Wada et al. reported improved survival in blunt trauma patients when WBCT was implemented in the secondary survey, but their instability criteria were not well-defined^31^. Blunt trauma patients lacking external indicators of potential internal injuries, WBCT may intuitively be more beneficial in the absence of a penetrating injury mechanism. In our matched subgroup analysis of blunt trauma patients however, we could not confirm this hypothesis. Another observational study by Fu et al. showed no significant difference in early mortality in haemodynamically unstable (defined as ≤ 90 mmHg SBP after 2000 ml fluid resuscitation) in abdominal blunt trauma patients^13^. Cook et al. also used propensity score matching to reduce confounding and described a reduced 24 h mortality, fewer urgent operations and more urgent angiographies in patients with abdominal trauma being positively examined with FAST and with SBP ≤ 90 mmHg on arrival and undergoing WBCT^14^. In a Colombian study by Ordonez et al., haemodynamic instability was defined as SBP < 100 mmHg, HR ≥ 100 bpm and transfusion of at least 4 units of packed red blood cells. In their study, unlike all others, the most frequent mechanism of trauma was penetrating. No significant difference in hospital mortality was observed^15^. A more recent paper from the same authors showed no difference in mortality between groups of progressively hypotensive patients undergoing a WBCT or not^32^. Tsutsumi et al. carried out two studies on the topic: a retrospective cohort study among hypotensive (SBP ≤ 90 mmHg) blunt trauma patients showed that despite ATLS recommendations, 92% of patients underwent WBCT, without increasing in-hospital mortality^16^. Their second registry-based analysis, including severely injured patients not solely based on haemodynamic criteria, showed a significant decrease in in-hospital mortality if WBCT was employed^17^. Sierink et al. found no significant difference in in-hospital mortality between patients undergoing WBCT versus on-demand selective CT in a randomised controlled trial (REACT-2). However, this study did not include haemodynamically unstable patients only^33^. A secondary analysis of the REACT-2 trial showed the same absolute decrease of mortality found in this study in patients requiring emergency bleeding control interventions^18^. Despite an important clinically significant difference, the result was not statistically significant due to a underpowered analysis like this present study. In all of these studies, haemodynamic instability was mostly defined by a low SBP, without taking into account any metabolic surrogates of shock, such as base excess and lactate. Therefore, the unstable patient groups in these studies (with only a few exceptions) may have included cases who were solely hypotensive (SBP < 100) but not in true shock with evidence of tissular hypoperfusion. In our study, only 53% (n = 161) of the 302 patients with a SBP < 100 mmHg had metabolic evidence of circulatory shock. The increasing use of WBCT in haemodynamically unstable trauma patients challenges the somewhat dogmatic view of WBCT as the "donut of death"^29^. Objective arguments against using WBCT in unstable trauma patients for fear of circulatory arrest from ongoing uncontrolled bleeding are scarce. Few data were found in the literature on the incidence of significant adverse events during the scanning phase. Ghafil et al. described an incidence of 47.9% for any adverse event, most frequently problems with IV access or hypotension, prolonging the WBCT duration by 9 min on average^34^. By contrast, in a subsequent matched-pair analysis based on data from the German trauma registry DGU^®^ (SBP upon ED arrival was the only matching criterion), head- or WBCT were more commonly performed among survivors, without any prolongation of time spent in the ED^35^. Even though WBCT remains reserved for haemodynamically stable trauma patients in the latest ATLS recommendations, the German 2022 polytrauma national guidelines decreased the minimal SBP to ≥ 60 mmHg as a limit to perform WBCT if no immediate therapeutic intervention is clearly indicated^36^.

As reflected by the results of the present retrospective analysis of our own practice, WBCT is being increasingly used in haemodynamically unstable trauma patients. However, it is important to note that, per se, WBCT can only be beneficial in case of immediate availability of a subsequent therapeutic option, either surgery or interventional radiology. This is an argument in favor of centralisation of severely injured patients in hospitals with 24/7 availability of the necessary infrastructure and expertise. Hybrid operating rooms, combining the simultaneous availability of whole-body CT, interventional radiology and surgery may be of particular benefit for severely injured patients^37^.

### Strengths and limitations

First, we used data from a well-designed inception cohort. The database included variables from the prehospital setting to the discharge from the hospital. Second, this study used an appropriate definition of haemodynamically unstable trauma patients. We selected a homogenous subset of patients to minimize the bias towards the null due to hypotensive patients not in circulatory shock. However, we cannot exclude a selection bias due to missing values or documentation errors in the trauma registry. The use of lactate and base excess in the patient selection limited the risk of misclassification. However, the retrospective study design presents a high risk of selection bias. Also, documentation bias precluded the inclusion of potentially confounding variables such as volumes and dosages of fluids, vasopressors and other drugs administered. Performing a randomised controlled trial in this setting is questionable due to ethical concerns. Third, we used rigorous methods of analysis with propensity-score matching to limit the influence of unknown confounders. We performed a multivariate model to control for known confounders. In addition, we performed IPW analysis as sensitivity analysis for the propensity score matching. All analyses showed the same results, which confirms a correct internal validity. Fourth, we cannot exclude measurement errors leading to regression bias due to the method of data collection. Fifth, due to its design, this study lacks statistical power, potentially leading to type II error. We cannot exclude a significant decrease in mortality with WBCT. Sixth, the generalisability is limited as this study is based on data from a single European trauma center. Transportability to different trauma system is therefore reduced. Finally, the study covers a period of more than 12 years. During this time, several minor structural and/or organisational changes in care have occurred, potentially influencing the outcomes.

## Conclusion

There was no statistically significant difference in mortality at 24 h among severely injured patients in circulatory shock, whether WBCT was used or not during the initial phase of management. This study indicates that WBCT may be offered to trauma patients in circulatory shock without jeopardizing early survival.

### Supplementary Information

Supplementary Information.

## Data Availability

The dataset supporting the conclusions of this article is available from the corresponding author on reasonable request.

## Abbreviations

ASA: The American Society of Anesthesiologists
ATLS: Advanced Trauma Life Support
BATT: Bleeding Audit and Triage Trauma Score
CT: Computed tomography
ED: Emergency Department
eFAST: Extended Focused Assessment with Sonography in Trauma
HR: Heart rate
ICU: Intensive care unit
IPW: Inverse probability weighing
ISS: Injury Severity Score
NCCLS: National Committee for Clinical Laboratory Standards
NISS: New Injury Severity Score
PS: Propensity Score
RCT: Randomised Controlled Trial
SBP: Systolic blood pressure
STROBE: Strengthening the Reporting of Observational Studies in Epidemiology
TRAC: Trauma Registry of Acute Care
WBCT: Whole body computed tomography
