# Supplementary figures and images for: The use of whole body computed tomography does not lead to increased 24-h mortality in severely injured patients in circulatory shock
Source: Sci Rep. 2024 Jan 25;14:2169. doi: 10.1038/s41598-024-52657-5 (PMC10810913; doi:10.1038/s41598-024-52657-5)

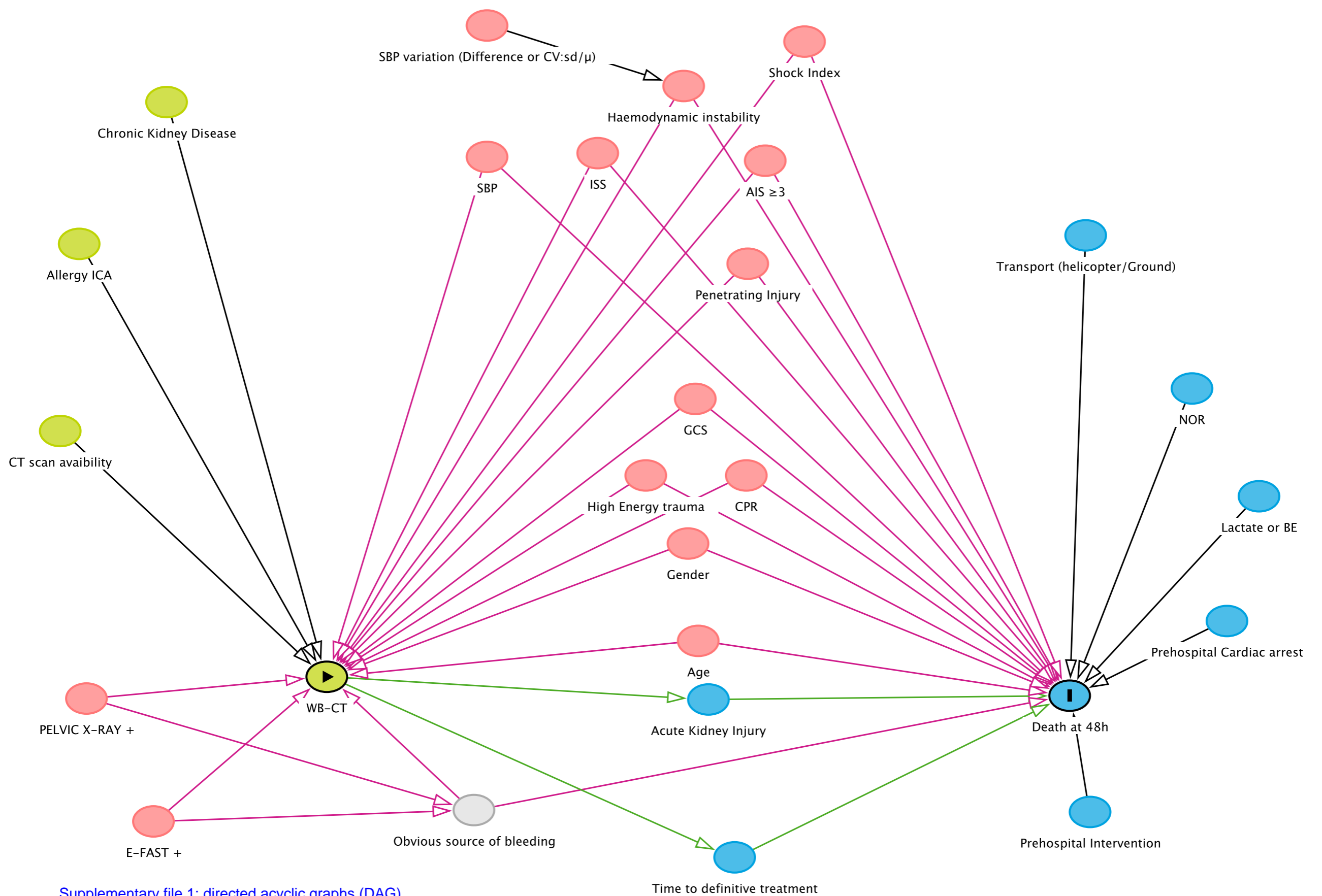

Supplementary file 1: directed acyclic graphs (DAG)

Supplement: Supplementary file 1 — Supplementary Information. [file 41598_2024_52657_MOESM1_ESM.pdf]
